# Conbercept and Retinal Photocoagulation in the treatment of Diabetic Macular Edema

**DOI:** 10.12669/pjms.35.6.512

**Published:** 2019

**Authors:** Weizhe Meng, Ronghua Li, Xiufen Xie

**Affiliations:** 1 Weizhe Meng, Binzhou People’s Hospital, Shandong, 256610, China; 2 Ronghua Li, Binzhou People’s Hospital, Shandong, 256610, China; 3 Xiufen Xie, Department of Intervention, The First Hospital Affiliated with Shandong First Medical University, 250014. China

**Keywords:** Conbercept, Retinal photocoagulation, Diabetic macular edema

## Abstract

**Objective:**

To explore the clinical efficacy of intravitreal injection of conbercept in combination with retinal laser photocoagulation in the treatment of diabetic macular edema.

**Methods:**

Ninety patients with diabetic macular edema were selected and grouped into an observation group and a control group using random number table, 45 patients (45 eyes) each group. The control group was given retinal laser photocoagulation, while the observation group was given intravitreal injection of Conbercept on the basis of panretinal photocoagulation. The Best Corrected Visual Acuity (BCVA), thickness of retinal nerve fibre layer (RNFL) and macular thickness were measured through relevant examinations before and after treatment. The intraocular pressures of patients in the two groups were evaluated, and moreover the complications were recorded.

**Results:**

The RNFL thickness and macular thickness of the two groups had no statistically significant differences before treatment (P>0.05) and decreased significantly after treatment; the decrease amplitude of the observation group was significantly larger than that of the control group (P<0.05). The BCVA of both groups significantly increased in the 1^st^, 2^nd^ and 4^th^ week after treatment (P<0.05); the increase amplitude of BCVA of the observation group was more significant than that of the control group at different time points after treatment (P<0.05). The intraocular pressure of the observation group was not significantly different with that of the control group in the 1^st^, 2^nd^ and 4^th^ week after treatment (P>0.05). There were no severe eye complications and systemic adverse reactions in both groups in the process of follow up.

**Conclusion:**

Intravitreal injection of conbercept in combination with retinal laser photocoagulation performs better in improving the BCVA and central macular thickness of patients with diabetic macular edema compared to retinal laser photocoagulation and has high safety.

## INTRODUCTION

With the improvement of people’s living standard, the incidence of diabetes has increased, and the number of diabetic patients has reached 420 million,^1^,^2^ A study found that the incidence of diabetic retinopathy (DR) in type 2 diabetes mellitus was about 35%.^3^ Diabetic macular edema (DME) is a common complication of DR and the primary cause of visual loss.^4^ The incidence of diabetic macular edema in DR patients was 7%.^5^ Laser photocoagulation is currently the main method for the treatment of diabetic macular edema.^6^ It can improve visual acuity and delay the decline of visual acuity. However, due to the destructive effect of laser itself on eye tissues, laser photocoagulation can cause severe visual impairment, vitreous hemorrhage and visual deformations, and not all DME patients who receive laser therapy will have edema relief. Therefore laser therapy has limitations.^7^

In recent years, it has been gradually confirmed that vascular endothelial growth factor (VEGF) plays an important role in the occurrence and development of DR.^8^,^9^ High expression of VEGF can lead to angiogenesis, increase vascular permeability, severely destroy blood retinal barrier (BRB), promote macular edema formation, and lead to irreversible visual damage.^10^ In the past ten years, reducing level of intraocular VEGF with intravitreal injection of anti-VEGF drugs has been widely used in clinical treatment, which can significantly relieve DME.^11^ Conbercept is a multi-VEGF multi-target fusion protein independently developed in China. It is a new type I biological drug, which provides a new therapeutic direction for the treatment of DME.^12^ In this study, 90 patients with progressive DR were evaluated by the clinical effect of intravitreal injection of Conbercept combined with panretinal photocoagulation was further analyzed taking 90 patients as subjects and retinal nerve fibre layer (RNFL) thickness, macular thickness and best corrected visual acuity (BCVA) as evaluation indicators.

## METHODS

Ninety patients with diabetic macular edema who were admitted to Binzhou People’s Hospital between August 2016 and October 2017 were selected, and ninety eyes were included. They were divided into an observation group and a control group. Patients who had history of type 2 diabetes mellitus, had normal blood glucose level and pressure, were diagnosed by fluorescence fundus angiography (FFA), conformed to the diagnostic criteria for DR recommended by American Diabetes Association (ADA),^13^ were diagnosed as macular oedema with clinical significance according to the standards of Early Treatment Diabetic Retinopathy Study, aged 40~80 years, had BCVA between 0.01 and 0.6 before treatment, had no history of retinal vein obstruction and glaucoma, and had DME in one eye were included. Those who received ophthalmologic operation such as fundus laser and intraocular injection, needed surgery because of large area of vitreous hemorrhage and retinal detachment, were pregnant or lactant, had severe allergic constitution, or had severe cardiac, hepatic and renal dysfunction were excluded. The observation group consisted of 45 patients (45 eyes), including 26 males and 19 females; they aged 43~80 years (average 63.57±6.73 years); the course of diabetes was 4~12 years (average (6.92±2.21) years). The control group consisted of 45 patients (45 eyes), including 28 males and 17 females; they aged 42~75 years (average (62.21±5.40) years); the course of diabetes was 4~11 years (average (6.88±2.14) years). There was no significant difference in sex constituent ratio, age and course of diabetes between the two groups before treatment (P>0.05). All patients enrolled in this study signed the informed consent, and the study was approved by the ethics committee of our hospital. (Approval letter August 12, 2018)

### Treatment methods

Patients in the control group received retinal laser photocoagulation. Local macular edema was given local retinal photocoagulation. Diffuse macular edema was given grid laser photocoagulation. 532 laser machine (ZEISS) was used. The parameters of the laser were: 110~250 mW spot energy, 50~300 μm diameter of spot, 0.1~0.15 s, and one spot diameter between photocoagulation spot. 200~500 points were treated every time.

Patients in the observation group were given retinal laser photocoagulation one week after the intravitreal injection of conbercept. The detailed operation was as follows. Eyes of patients were disinfected before surgery, and then the surface of eyes was anesthetized using 0.25% tetracaine solution. After anesthesia, the eyelid was held apart. The conjunctival sac was washed using tobramycin diluent. Then a 1 mL disposable sterilized syringe which was equipped with 29 G special needle was used to extract conbercept injection (Chengdu Kanghong Biotechnology Co., Ltd., China, SFDA approval number: S20130012) which was prepared in advanced and the site where was below the temple and 4 mm behind limbus of sclera was punctured; the needle tip was inserted to the vitreous cavity. Then 0.05 mL of conbercept injection was slowly injected into the vitreous body. The syringe needle was pulled out after injection. The needle eye was mildly pressed by cotton swab for 1~2 minutes. The eye was patched using tobramycin and dexamethasone eye ointment and given antibiotic drops for three days. Intraocular pressure was measured by regular reexamination.

Patients in both groups were given nursing intervention. The first one was mental nursing. Some patients felt depressed because of insufficient understanding of disease, impaired vision and limited activity of daily living. Therefore patients were informed with knowledge about disease, method, objective, efficacy and procedures of treatment and matters needing attention in the process of treatment and cases cured after treatment to eliminate their anxiety and fear. Next was postoperative nursing. Patients were asked not to rub the operative eye with hands as eye rubbing could cause corneal injury because of the application of topical anesthetic, Alcaine. Eyes were given antibiotic drops for three days, four times each day. The vision of the operative eye might be blurred because of complete mydriasis before photocoagulation; patients were informed that such a blurred vision phenomenon was temporary and would disappear after 6~8 hours. Patients were reminded of vision examination, fundus reexamination and FFA. Last was blood glucose control nursing. For patients with diabetes, surgical stress response may lead to increased secretion of glucagon, which can result in further increase of blood glucose. It can weaken wound healing capability and increase risks of infection. Therefore monitoring of blood glucose was strengthened, and guidance for diet and application of hypoglycemic agent were enhanced to promote wound healing and avoid occurrence of complications.

### RNFL thickness and macular thickness examination

Optical coherence tomography was performed before treatment and seven days after treatment using Germany Heidelberg optical coherence tomography. Retina around the optic disk was given circular scan to obtain the average RNFL thickness of upper and lower sides of the optic disk, temple and annular area of nose and 360° RNFL thickness. (2) Vision: BCVA was measured using the international standard visual acuity chart before treatment and one, two and four weeks after treatment and transformed into Log MAR vision according to reference.^10^ (3) Intraocular pressure was recorded before treatment and one, two and four weeks after treatment. (4) Complications: Complications such as corneal edema, anterior chamber inflammatory reaction, high intraocular pressure, retinal hemorrhage, neovascular glaucoma and endophthalmitis were observed 3 months after treatment.

### Statistical Analysis

The research data were processed by SPSS 21.0. Categorical data were expressed as rate (%) and processed by Chi-square test. Measurement data were expressed as Mean± SD and processed by paired or independent t test. Difference was considered as statistically significant if P<0.05.

## RESULTS

There was no significant difference in the RNFL thickness and macular thickness between the two groups before treatment (P>0.05); but the RNFL thickness and macular thickness significantly decreased after treatment. The average RNFL thickness of upper and lower sides of the optic disk, temple and annular area of nose, 360° RNFL thickness and macular thickness of the observation group were significantly lower than those of the control group, and the difference had statistical significance (P<0.05, Table-I).

**Table I:** RNFL thickness and macular thickness between the two groups (Mean±SD).

Groups		Observation group		Control group	

		Prior treatment	Post treatment	Prior treatment	Post treatment
RNFL thickness (μm)	Upper side	164.13±28.78	125.24±19.02	162.35±24.87	142.23±19.18
	Lower side	190.33±28.42	134.42±25.12	193.30±25.58	155.28±27.27
	Temple	94.36±9.02	70.61±9.03	95.33±9.73	76.34±8.45
	Annular area of nose	116.43±16.84	83.13±9.28	111.33±14.59	89.38±10.68
	360°	164.40±210.39	108.95±17.81	166.88±20.73	120.84±18.33
Macular thickness (μm)		373.57±44.21	239.66±20.73	370.31±40.82	249.44±26.93

There was no significant difference in the BCVA between the two groups before treatment (P>0.05). The BCVA of both groups significantly improved after treatment. The increase amplitude of BCVA of the observation group was significantly larger than that of the control group in the 1^st^, 2^nd^ and 4^th^ week after treatment, and the difference had statistical significance (P<0.05, Table-II).

**Table II:** BCVA between the two groups before and after treatment (Mean±SD, log MAR).

Group	Observation group	Control group
Prior treatment	0.07±0.02	0.07±0.01
1^st^ week after treatment	0.27±0.05*^#^	0.20±0.04*
2^nd^ week after treatment	0.29±0.07*^#^	0.23±0.05*
4^th^ week after treatment	0.30±0.07*^#^	0.26±0.05*

***Note:***

* indicated that P<0.05 compared to before treatment within the same group;

# indicated that P<0.05 compared to the control group.

The intraocular pressure of the two groups at the 1^st^ and 2^nd^ week after operation was significantly higher than that before treatment (P<0.05), but there was no significant difference between the intraocular pressure at the 4^th^ week after treatment and that before treatment (P>0.05). There was no significant difference in the intraocular pressure between the two groups at different time points (P>0.05, Table-III).

**Table III:** Intraocular pressure between the two groups before and after treatment (Mean±SD, mmHg).

Group	Observation group	Control group
Prior treatment	12.35±2.13	12.40±2.30
1^st^ week after treatment	15.56±2.08*	15.90±2.3*
2^nd^ week after treatment	13.17±2.04*	13.72±2.09*
4^th^ week after treatment	12.13±1.96	12.46±2.12

***Note:***

* indicated P<0.05 compared to before treatment within the same group.

During the follow-up period, no severe eye complications such as such as corneal edema, anterior chamber inflammatory reaction, retinal hemorrhage, neovascular glaucoma and endophthalmitis and systemic adverse reactions occurred in both groups. Only one patient in the observation group had transient intraocular hypertension (1.11%), which was relieved 1-2 days after treatment without special treatment.

## DISCUSSION

DME is one of the main causes of vision loss in DR patients, which can seriously affect their visual function and quality of life. Ting et al. made Meta-analysis on 22,896 diabetic patients and found that the prevalence of proliferative DR and DME was 6.96% and 6.81% respectively.^14^ The pathogenesis of DME is complex. A study has shown that it may be related to basement membrane thickening,^15^ cell apoptosis and fluid accumulation in the macular area caused by protein and water entering the retinal parenchyma because of increase of blood sugar level, which is manifested as macular edema. DME develops slowly. Most patients lack correct understanding of DME, delaying the best treatment opportunity and eventually leading to irreversible visual impairment.^16^

At present, laser photocoagulation is the most commonly used method for the treatment of DME. It can reduce the oxygen consumption of retina through generating grey white or white laser scar based on thermal energy.^17^ But Zhang et al.^18^ found that the effect of laser treatment weakened as time went on and laser treatment might damage photoreceptor and blood capillary and result in temporary loss of vision and increase of retinal thickness. Since neovascularization and/or fibroplasia are the main characteristics of DR, it can lead to abnormal color perception, reduced contrast sensitivity and RNFL thickness changes. In order to further improve the therapeutic effect of DR and improve the quality of life of patients, many researchers unanimously advocate the combination of intravitreal injection of anti-VEGF drugs and laser photocoagulation.^19^,^20^

VEGF is a potent and disseminable mitogen, which can specifically bind with VEGF receptor on the surface of vascular endothelial cells to stimulate the proliferation, differentiation, migration of vascular endothelial cells and lumen formation. A clinical study showed that intravitreal injection of anti-VEGF drugs could effectively reduce DME and improve visual acuity. In addition,^21^ some randomized, double-blind, controlled phase III clinical studies on anti-vascular drugs showed that anti-vascular therapy was better than laser alone in promoting visual acuity recovery and the average improvement of visual acuity in the anti-vascular drugs group was better than that in the laser alone group.^22^,^23^ Conbercept ophthalmic injection is the first anti-VEGF drug independently developed in China. Conbercept ophthalmic injection is a fusion protein of VFGF receptor and recombinant human immunoglobulin Fc gene. It can competitively inhibit the binding of VEGF and receptor and the activation of VEGF family receptor, thereby inhibiting the proliferation endothelial cells and angiogenesis and alleviating macular edema.^24^ The results of this study showed that the RNFL thickness and macular thickness of the two groups had no significant differences before treatment, the values of the two indexes significantly decreased in both groups after treatment, the decrease amplitude of the observation group was larger than that of the control group, and the increase amplitude of BCVA in the observation group was more significant than that in the control group at the 7th, 14th and 28th day after treatment, which was consistent with the results of Xie et al.^25^ The reason might be that intravitreal injection of conbercept could inhibit the formation and development of neovascularization, alleviate vascular leakage, restore retinal transparency, and reduce the demand for high laser energy, and moreover laser therapy could seal capillaries to reduce leakage, thereby reducing the oxygen consumption of the outer retina.

In addition, the study of intraocular pressure in the two groups after surgery found that the intraocular pressure of the two groups were within the normal range, and there was no significant difference between the two groups (P>0.05), indicating that conbercept ophthalmic injection did not affect aqueous fluid circulation and increase intraocular pressure. During the follow-up period, severe eye complications such as corneal edema, anterior chamber inflammatory reaction, retinal hemorrhage, neovascular glaucoma and endophthalmitis and systemic adverse reactions did not happen in the two groups, which indicated that the combination of conbercept and laser photocoagulation was safe and tolerable.

## CONCLUSION

Intra vitreal injection of conbercept combined with laser photocoagulation can significantly improve visual acuity and macular central thickness and restore retinal structure in a short time in the treatment of DME, which is better than retinal laser photocoagulation alone. However, the clinical treatment should consider patients’ systemic and economic conditions. The follow-up time of this study was relatively short and more middle and long-term clinical observations of subsequent development of disease course and curative efficacy are needed.

### Authors’ Contribution:

**WZM & RHL:** Study design, data collection and analysis.

**WZM & XFX:** Manuscript preparation, drafting and revising.

**WZM:** Review and final approval of manuscript.
